# A qualitative study of a primary-care based intervention to improve the management of patients with heart failure: the dynamic relationship between facilitation and context

**DOI:** 10.1186/1471-2296-15-153

**Published:** 2014-09-18

**Authors:** Stephanie Tierney, Roman Kislov, Christi Deaton

**Affiliations:** Royal College of Nursing Research Institute, University of Warwick, Coventry, UK; Manchester Business School, University of Manchester, Manchester, UK; Florence Nightingale Foundation Professor of Clinical Nursing Research, Department of Public Health and Primary Care, University of Cambridge School of Clinical Medicine, Cambridge, UK

**Keywords:** Evidence-based practice, Knowledge mobilisation, Qualitative research, Semi-structured interviews, Heart failure, Facilitation

## Abstract

**Background:**

There is currently a growing emphasis in primary care on upscaling the provision of evidence-based services for specific conditions, such as heart failure (HF), which have traditionally been seen as part of a specialist’s domain. While contextual challenges associated with improvement in primary care have been documented previously, we still know relatively little about how the intentional, theory-informed facilitation of evidence-based change is shaped by contextual factors within this healthcare setting. Hence, a qualitative study was conducted to address the question: How is the process of facilitating evidence-based practice affected by the context of primary care?

**Methods:**

Data collection took place across general practices in northwest England as part of a process evaluation of the Greater Manchester HF Investigation Tool (GM-HFIT) - a programme of work aiming to improve the management of HF in primary care. Semi-structured interviews, with purposefully selected GM-HFIT team members (n = 9) and primary care practitioners (n = 7), were supplemented by observational data and a three-month diary reflecting on facilitation activities. Framework analysis was used to manage and interpret data.

**Results:**

We describe a complex and dynamic interplay between facilitation and context, focusing on three major themes: (1) Addressing macro and micro agendas; (2) Forming a facilitative unit; (3) Maintaining momentum. We show that HF specialist nurses (HFSNs) have a high level of professional credibility, which allows them to play a key role in making recommendations to practices for improving patient care. At the same time, we argue that contextual factors, such as top-level endorsement, the necessity to comply with a performance measurement system, and the varying involvement of practice nurses produce tensions that can have both an enabling and constraining effect on the process of facilitation.

**Conclusions:**

When facilitating the transfer of evidence, context is an important aspect to consider at a macro and micro level; a complex interplay can exist between these levels, which may constrain or enable efforts to amend practice. Those involved in facilitating change within primary care have to manage tensions arising from the interplay of these different contextual forces to minimise their impact on efforts to alter practice based on best evidence.

## Background

The role of primary care has been extended in the past two decades, with increasing emphasis on chronic disease management [1]. General practitioners (GP) and practice nurses (PNs) are expected to provide evidence-based treatment for patients with conditions such as heart failure (HF), and limit referral to specialists. HF is a complex syndrome, consisting of signs and symptoms that imply there is a problem with the heart’s ability to act as a pump [2]. It is defined as a primary care-sensitive condition because it is believed that improving GP management reduces the need for emergency admissions [3]. Despite guidelines for evidence-based management [2, 4], research suggests care for HF often remains suboptimal [5–7]. This may stem from the management of a predominantly older group, with concomitant comorbid conditions, and from deficits in the organisation of care, such as poor communication between providers [8–10].

One way of improving management for this complex problem is by using methods and frameworks developed in the emerging field of implementation science. For example, the PARiHS (Promoting Action on Research Implementation in Health Services) Framework [11] reflects the complexity in which the transfer of evidence into day-to-day clinical practice occurs [12]. It consists of three key interactive elements: *evidence* (research, clinical experience and patient preference), *context* (culture, leadership and measurement) and *facilitation* (characteristics, role and style) [13]. Evidence alone is insufficient as it needs to be translated and particularised in the context of caring for individual patients [12, 14]. Being sensitive to context requires facilitation by an individual with specific attributes and knowledge. Facilitation should be seen as a distinct implementation initiative [15], ranging from providing help and support to achieve individual goals to enabling teams to analyse, reflect, and change their own attitudes, behaviours, and ways of working [11].

Although a plethora of work has been conducted on the topic of implementing evidence into practice, there is a gap in understanding how to promote change in specific contexts, particularly in primary care practices, which can be seen as independent businesses with significant autonomy in day-to-day operations [16]. We present a qualitative process evaluation of a multifaceted project underpinned by the PARiHS framework - the Greater Manchester Heart Failure Investigation Tool (GM-HFIT). GM-HFIT (http://clahrc-gm.nihr.ac.uk/our-work-2008-2013/gm-hfit/) aimed to: a) ensure care of HF was consistent with national guidelines, b) advance knowledge and skills of primary care staff, c) improve quality of data held in primary care, and d) increase the number of patients receiving optimum medication [17]. The project was novel in its focus on primary care management of HF, the utilisation of implementation science knowledge in its design, delivery and evaluation, and the fundamental role of heart failure specialist nurses (HFSNs) in achieving its objectives.

## Aim

The purpose of this study was to describe and explore the process of facilitating evidence based practice in GM-HFIT within the context of primary care.

## Methods

### Design

A qualitative methodology was selected to explore experiences and meanings, whilst remaining sensitive to the social context. A qualitative descriptive study [18, 19] was conducted to provide an understanding of processes associated with facilitation. This approach is not directed by a specific philosophical assumption in the form of one of the known qualitative methodologies [20], but can be employed when exploring perspectives of a process.

### Setting

GM-HFIT was developed by members of the Collaboration for Leadership in Applied Research and Care for Greater Manchester (GM CLAHRC) in collaboration with clinicians and managers to address a number of challenges in managing patients with HF in primary care. The GM-HFIT team included knowledge transfer associates and managers (KTAs) and HFSNs. KTAs were appointments made specifically to CLAHRC [21]. Their role was to facilitate implementation initiatives within healthcare settings by working across professional and organisational boundaries. One of the KTAs we interviewed had a clinical background; the rest were non-clinicians with an interest in knowledge transfer. HFSNs were not part of primary care practices, and most were seconded from secondary care and specialist services.

GM-HFIT consisted of *case finding* (identifying unrecorded HF or potential HF cases), *register verification* (ensuring the accuracy of registers), and *audit* of evidence-based indicators of care. Members of the team conducted the work within individual GP surgeries and arranged a feedback session to discuss recommendations with staff. Interactive small group education was offered to practices, as well as informal training at the surgery’s request. Group education covered the management of HF: diagnosis, monitoring and treatment, medication titration, lifestyle and self-management, palliative care. To facilitate audit feedback, a traffic light system was created to show the percentage of patients meeting indicators of care on the audit. The total traffic light score ranged from Gold (outstanding care), to Green (high standard of care), Amber (good care but areas for improvement) and Red (falling short of optimum care and requiring major improvements). Re-audit of practices was performed 6-9 months after the initial investigation. The project commenced in one location (10 practices) in 2010 and was rolled out into two other areas (total 35 practices) during 2012 - 2013. Facilitation was considered in broad terms and included establishing rapport with practices at the start, arranging meetings to acclimatise to the setting and to discuss findings with staff, and providing education and support.

### Sample

A purposive sample was composed of key informants chosen to represent a range of views based on their involvement in GM-HFIT. It included those with responsibility for designing, delivering or receiving the intervention. These individuals were approached by either the first or second author, who attended meetings run by the GM-HFIT team. We also used snowball sampling, via KTAs, to recruit staff from a range of practices. Recruitment continued until we had a diverse range of individuals in terms of role in GM-HFIT, and staff from all three locations where this intervention was carried out. Practices were at different stages in facilitation, which provided variation in data. Formal ethical approval was not required for the study since it was classified by National Health Service (NHS) governance procedures as service evaluation. However, all participants received an information sheet and consented to the recording of their interview and use of their data.

### Data collection

Semi-structured interviews were conducted with individuals or small groups (2-3 people). Groups were composed of individuals with the same job title (nurses or GPs), to avoid potential power issues. Topic guides were developed in advance, through discussion among members of the research team. They included broad, open ended questions, such as:

- What has been your role in GM-HFIT?
- What were you hoping GM-HFIT would do?
  - ◦How well do you think it achieved its aims?
  - ◦What worked?
  - ◦What did not work?
  - ◦What could be improved?

- Which parts of GM-HFIT do you think are most important for introducing change in HF care in primary care?
- What do you feel patients will have noticed as a result of GM-HFIT?
- Who are the key players in making GM-HFIT work?

The topic guide was used to focus the discussion but, in line with the flexible nature of qualitative research, other issues were explored if raised by participants and relevant to the study’s aim. Semi-structured interviews enabled participants to give their own accounts and to reveal what they thought were important process factors. Interviews were conducted between April-August 2013 by the first two authors who were not involved in designing or delivering GM-HFIT. They took place in a university office, hospital setting, primary care practice or by telephone, whichever was most convenient for participants. All interviews were audio-recorded and transcribed verbatim.

To enhance our understanding of the interview data, we consulted notes made by one of the authors (RK) during direct observation (39 hours) of various GM-HFIT activities (e.g. implementation team meetings, learning sessions, practice visits). In addition, the lead HFSN kept an electronic record of facilitation activities over a 3 month period, which was shared with the researchers. After charting interview data, we considered information from these two sources, which supported rather than altered any of the themes identified already from the analysis of interview transcripts.

### Analysis

Framework analysis was used to categorise data [22], progressing through the following stages: 1) Familiarisation with the data; 2) Development of a thematic framework; 3) Indexing data; 4) Devising a series of thematic charts; 5) Mapping and interpreting data. As interview transcripts were produced they were read by the authors who made notes of emerging codes. They came together to share their ideas and to decide on an indexing scheme, which was applied to all transcripts by the first author to chart data. At another team meeting, these charts were discussed by the authors and considered alongside observational and diary data to produce final themes. Table 1 illustrates how we moved from initial, broader categories to final themes. NVivo9, a qualitative software programme, expedited organisation and coding of data and helped to identify exemplars and representative quotations.

**Table 1:** **An example of how a theme evolved from categories and sub-categories**

Theme	Forming a facilitative unit								
Sub-theme	***Flexible and evolving approach***		***Complementary team roles***				***Presenting messages about change***		
**Category**	*Development of GM-HFIT*		*Roles and responsibilities*				*Presentation of results*		
**Code**	Evolving	Flexibility	SNs’ role	KTs’ role	Primary care staff’s role	Joint enterprise	Depth of work	How data are presented	Traffic lights system

## Results

We interviewed a diverse range of individuals (n = 16) who were involved in GM-HFIT as providers or recipients (see Table 2). Interviews lasted 30-80 minutes.

**Table 2:** **Details of interviewees and interviews**

Job title	Number	Length of interviews	Where interviews took place
Knowledge transfer professional (KTA)	5	60-80 minutes	Hospital or University
Heart failure specialist nurse (HFSN)	4	45-65 minutes	University or GP clinics
General practitioner (GP)	3	30-35 minutes	GP clinics or over the phone
Practice nurse (PN)	4	35-45 minutes	GP clinics or over the phone

Following close examination of all data, three final themes emerged, which are described below along with their constituent sub-themes. Data highlight that facilitation had to be multidimensional and adaptable to local circumstances, and support practices in taking responsibility if changes were to be sustainable. Anonymised quotations from interviewees are included to support points raised.

## Addressing macro and micro agendas

Changing practice calls for a focus extending beyond the individual by considering wider influences [12]. In our data, those wishing to engender change had to contemplate and respect macro (e.g. NHS initiatives and regional organisations) and micro (e.g. individual clinicians and practices) factors as each could impact on motivations to engage with GM-HFIT. Motivation to take part in the project could be seen as an essential first step in enabling facilitation.

### National level

Targets for delivery of care were repeatedly referred to during interviews as a reason to get involved in GM-HFIT. Primary care practices are financially incentivised to achieve four indicators of care for HF as part of the Quality Outcomes Framework (QOF). When introducing the project to practices, the GM-HFIT team highlighted how it could help with case finding and register accuracy, thereby improving QOF scores. The QOF indicators were mentioned by several interviewees:

PN3: *“…we don’t like to go on about QOF but at the end of the day you need a certain percentage, or that’s what they try to look at, that you’ll have so many percent in the population, you know, on your records and so it’s to try and get that up to speed as well and just make sure that nobody slips through the net…”*

It was noted that QOF scores for HF were limited:

HFSN2: *“…[QOF] did have 4 quality indicators, maintain a register, confirm it with an echo or specialist assessment and then make sure they’re on an ACE or beta-blocker… So it never really asked for anything else. It never said do an annual review. It never said check a pulse or blood pressure…”*

This lack of congruence between QOF targets and guidelines for best practice in HF could make facilitation efforts aimed at sustaining change difficult.

### Local level

Motivation to be involved with GM-HFIT was strengthened in some cases by support from an influential, regional organisation. In one location, surgeries were recruited from a well-established collection of practices and in another the local commissioning group championed the project. This regional endorsement was seen as contributing to an excellent uptake of GM-HFIT. Part of the reason for this positive response may have been a competitive element between practices and a fear of lagging behind in terms of service delivery:

GP2: *“If we didn’t do that [take part in GM-HFIT], we might have been the only practice in [area] that didn’t take part in it and then of course say you go to meetings and we do have more patients being admitted well it would be because you didn’t take part in the audit. You have to be seen to be progressing.”*

Support from an influential body was not present in the first location involved in GM-HFIT, where engagement was described as more difficult. However, involving higher level bodies increased demand for GM-HFIT, leading to capacity problems in terms of getting work done in time:

KTA3: *“We wanted to work with 17 practices but then the [commissioning group] said that they wanted it offered out to all practices. So it kind of really doubled the amount of work, you know, that we need to do…and it’s so hard to find people to actually do the audits.”*

### Practice level

Individual practitioners were reported to vary in their response to GM-HFIT. Some were extremely interested in HF and its management but others listed it as just one of many conditions they had to address. In general, GPs took a lead on the project within a surgery, seeing it as mainly related to medication optimisation. Certain interviewees, therefore, suggested that audit feedback sessions were probably not suitable for administrative staff or nurses:

GP1: *“You see this programme’s basically a doctor based programme. I don’t think the staff nurse can initiate medication, like this type of medication or can arrange an echocardiogram based on her knowledge…”*

However, some PNs did take a lead:

KTA3: *“One practice nurse…we went to see her and she was really keen to do the project, so she led it…when we went back to re-audit she’d done lots of work…I think she actually requested echoes for every patient…she did everything she could and then she passed what she couldn’t do to the GPs but she probably acted as a facilitator because she pushed the GPs to do the work, rather than us going she kind of made sure they did what they needed to do.”*

Other PNs depicted their role in GM-HFIT as more technical and peripheral. They were asked to follow-up specific patients’ paperwork but did not feel engaged in the project:

PN4: *“I didn't even know they were even doing it in our practice…it had been arranged with our practice manager and GPs I believe… the project was presented to us at the very end with recommendations of changes that the practice should make.”*

## Forming a facilitative unit

Facilitation comes in differing formats, from helping practitioners to achieve a particular goal to empowering them to reflect on their own behaviours and ways of working [13]. Our study showed that over time facilitation became more tailored to the individual needs of a practice and training included a broader range of topics related to the types of patients seen in primary care (e.g. older, often with preserved ejection fraction). This shift in emphasis reflected a growing confidence and a better understanding of the role each member of the GM-HFIT team played in its execution.

### Flexible and evolving approach

Prior to developing GM-HFIT, a sustained period of consultation with key stakeholders ensued to consider how best to improve care for patients with HF. This resulted in the basic tenants of the project. However, GM-HFIT evolved due to needs of specific practices and attempts to improve systems. For example, initial case-finding searches were refined to become more time efficient. Education also became more tailored and interactive. PN2 recalled how HFSNs came to talk to her and other nursing colleagues, which was well received:

PN2: *“…we’ve not had an awful lot of updating on heart disease and on heart failure particularly…we all felt as though it was good, someone to come in and talk to the team…they do it from a nursing perspective…they speak the same [professional] language.”*

Despite adopting an overall flexible stance, to ensure that tasks were completed, attempts were made to standardise certain components of GM-HFIT. This was accepted by members of the team as a way of providing some constraints on how the project progressed:

HFSN3: *“[Lead HFSN] wanted to formalise and standardise the way we responded and wrote to the GPs…I'd already written in a certain style that suited me…then it was decided that they would standardise everything, so I've had to go back to all my original work and re-write it out so that it's in a standardised format…”*

### Complementary team roles

Members of the GM-HFIT team had a broad set of administrative and clinical skills, although interviewees noted that it could be difficult recruiting enough HFSNs due to specialist services being reluctant to second staff away from their clinical duties and the work itself being different from the usual role of these practitioners. KTAs were defined as experts in project management and understanding the broader NHS, whereas HFSNs were specialists in treating HF. KTAs dealt with co-ordinating the project and solving problems that impeded the work. HFSNs’ knowledge could then be channelled into advising on patient care and making clinical decisions when auditing registers. Setbacks with computer systems, described by interviewees as a common barrier to conducting audits, called for problem-solving skills from KTAs and a good rapport with practices to be able to modify where data collection took place:

KTA4: *“…at least two practices, the computer systems have crashed whilst we’ve been either in there or about to do some work…there’s been like a system wide crash of certain systems. There’s one practice that moved from one practice system to another and lost all of the relevant heart failure data…So I had to contact another practice and get us started there immediately that day…even though we weren’t set to start there for quite a while.”*

### Presenting messages about change

KTAs tended to produce the feedback document following an audit whilst HFSNs delivered its messages to practitioners. Their clinical expertise provided weight to recommendations for changes to care:

KTA5: *“…we were a bit sceptical at the beginning as to how that would work and whether we needed to get the GPs with specialist interest to go in and do the feedback…but that never quite came off …because the specialist nurses do have that knowledge …there’s not been an issue with that sort of peer to peer where you did think there might be a GP thinking oh, I’m the clinician, I make the decision, you’re just the nurse…”*

Practitioners commented on the depth of activity conducted by the GM-HFIT team. In some cases they had not been aware of the amount of work undertaken until results from the audit were presented at a feedback session:

GP2: *“When we saw the folder we thought wow they’ve gone to town…initially it was a bit daunting, you think, how do I understand this part here…how do we read the results, so when she [KTA] came we were reassured, ‘no you’ve only got to look at this part and that part etc.’”*

GM-HFIT team members emphasised that it was their role to provide feedback in a way that made practitioners feel supported, not criticised. The traffic light system enabled practices to see at a glance areas they needed to target:

KTA2: *“I think that was one of the biggest winners … what we did see is that they liked that clear information. Green – great, red – rubbish, simple as really, amber – have a look in a bit more detail and you’d find people flicking through looking for red ones and for amber ones…”*

The process was not limited to giving feedback and then leaving practices on their own; primary care staff were assisted in bringing about a change by the GM-HFIT team and helped to particularise evidence for individual patients:

KTA1: *“…we give them a pack, which is kind of the work they then need to do and in one of our feedback meetings one of the GPs said would you be able to sit with his colleague who’s going to lead it…just to give him the confidence to get him going…So [HFSN] went and sat with him, he blocked out a whole afternoon session this GP and [HFSN] went and sat with him for the first couple of hours and just kind of discussed cases.”*

## Maintaining momentum

Work about behavioural change shows that due to the pull of forces of resistance, it may be tempting to revert to previous practices, if maintenance strategies are not enacted [23]. Having enthusiastic personnel within a practice and staff with the requisite skills were seen as important for sustaining change, as was the use of reviews.

### Someone to carry the work forward

Attendance at the audit feedback influenced responsibility for leading work in a practice afterwards. Sometimes everyone, including administrative staff, came along, whilst in other cases just GPs were present. Generally, there was a discussion about who would take the work forward, resulting in an individual or individuals being nominated. Having a lead person within a practice for the project was felt to be a crucial step in its success:

KTA4: *“…I think it’s important that someone picks up the overall kind of lead for it because as I say otherwise the likelihood is that no-one will pick it up cause they’ll wait for someone to do something on it.”*

Proactive teams were described by KTAs as those that made a clear plan for how the work was to be carried out. In contrast, PN3 remarked that she and her colleagues were given a folder to address queries relating to patients on the register but they had not been allocated time to complete this work; hence, the folder remained on the shelf:

PN3: *“There’s been no time put aside for us to follow through what’s been done and that’s the frustrating bit really cause 70% of the work’s been done but there’s still that chunk that we’ve got to do.”*

### Up-skilling of staff

To be able to take the work forward staff needed to feel confident regarding the evidence and how to implement it. Education was important in this regard:

KTA2: *“…we talked to a GP…he openly said that when he trained…he was told never to prescribe beta blockers for heart failure cause at the time it was contraindicated… So he had never prescribed them because of what he was told and he hadn’t really updated himself, which is quite worrying but also quite encouraging that the education actually hit home and made the point.”*

GM-HFIT education was based on adult-learning principles [24], which involved starting from the needs of the individual, accepting that adult learners are goal-orientated and drawing on their wealth of professional and life experience. The GM-HFIT team commented that more focus could be placed on education sessions specifically for PNs, given that they often see patients for annual reviews. Yet the complex nature of HF could make PNs reticent about getting involved. Interviewees also noted that these nurses struggled to get time out of practice for educational purposes, especially if the education was not directly QOF-related. PNs suggested that any training should be delivered at the right pitch, preferably without GPs present:

PN1: *“…you’re not frightened if it’s other nurses to ask something that might appear to be a daft question, whereas you might keep quiet if you thought there were GPs or someone a bit more knowledgeable.”*

In education sessions, doctors were described as being interested in learning about medication, not really enquiring about self-management strategies, although there were some exceptions:

KTA3: *“…one of the GPs …asked all his patients to start doing daily weights and to contact him if they put on a certain amount of pounds over a certain period…What I would hope is that the patients, through the education that we give to the practices, that would be passed on so that patients are kind of maybe able to manage a bit more self-care, a bit more awareness of the signs that the condition’s worsening…”*

To support practitioners in providing information to patients, colourful leaflets on aspects of self-care were developed by the GM-HFIT team with patient and provider input.

### Electronic reminders

Follow-up of patients on a regular basis was seen by the GM-HFIT team as an important means of sustaining their work, but they were realistic that introduction of a template for this purpose may take time to implement:

KTA1: *“There are certain practices we know never in a month of Sundays will do that but there’s a good maybe 20% we know will and are engaged and we’ve tried to develop these templates on the computer to help them do [appropriate follow-up]…”*

They acknowledged that primary care staff may struggle to fit reviews into their working day and developed a guide to help practitioners with this task. Yet the limited requirements for HF management within QOF meant that implementation of a 6 monthly review, as recommended in HF guidelines [2, 4], could still be problematic:

HFSN1: *“…one GP said to me last week there’s no incentive to do heart failure reviews…he was talking in terms of remuneration because of the QOF points… It doesn’t go much further than that [QOF], whereas the template that’s been developed that we’ve tried to base on NICE goes a lot further.”*

## Discussion

Our research advances understanding of facilitating change in primary care specifically, and health settings more generally, by analysing data from a range of sources engaged in GM-HFIT. This intervention was based on principles shown to be effective in changing practice, including interactive education, audit and feedback, outreach and reminders [14]. GM-HFIT was novel in embracing a number of general practices of varying size, using different computer systems, and composed of personnel with diverging levels of enthusiasm and understanding of HF.

**Figure 1:**
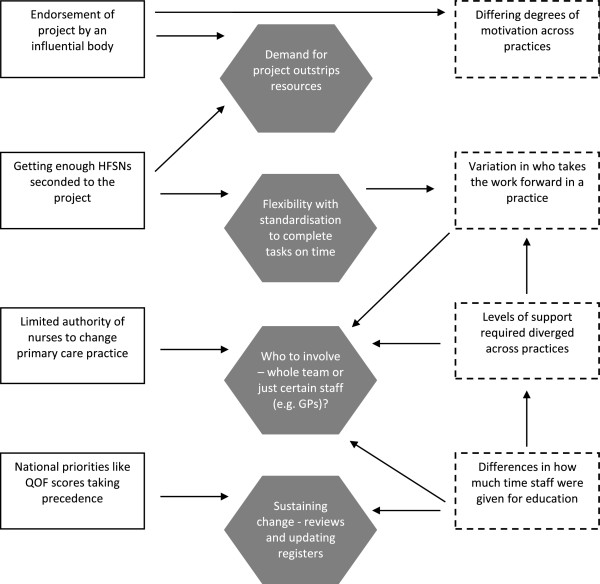
**Shows how macro and micro contextual factors contributed to tensions associated with facilitation in GM-HFIT** ***(key: rectangle with full line = macro context; rectangle with dashed line = micro context; shaded hexagon = project tensions)*** **.**

Our data reflect and extend aspects present within the PARiHS framework. For example, findings highlight the complex and dynamic nature of changing practice. We found that even if individuals were receptive to new evidence, they may not carry out work required for a project like GM-HFIT due to organisational pressures and barriers, such as lack of time, which has been noted in other studies [12]. Facilitating change was shaped by contextual factors and could result in some tensions. For example, endorsement of GM-HFIT from regional bodies assisted with recruitment of practices but placed a demand on resources. Likewise, how practices were encouraged to participate (some only doing so due to such promotion from an influential organisation) could lead to misunderstanding about GM-HFIT’s aims. Hence, findings emphasise contextual tensions that are present when trying to facilitate change in healthcare settings (Figure 1). Tensions differ from barriers described by others [14]. They are more rounded, with positive and negative components; they are less black or white than a barrier, emphasising relationships between stakeholders and the interplay between different contextual levels. Part of the role of facilitation appeared to be negotiating these tensions so they did not impede the project’s progression. Data suggest that responsibility for continuing the work was not always accepted by primary care staff because of the mixture of internal and external tensions (Figure 1). For example, national targets influenced what activities were accepted and adopted within a surgery. Similarly, tensions could emerge if individuals were unclear why the work was necessary. As a consequence, some team members may be less accommodating to change (e.g. delay following up queries from the audit).

Another tension to transpire in our data, which illuminates our understanding of facilitation in the PARiHS framework, was the requirement for flexibility within GM-HFIT at the same time as some degree of standardisation to ensure the work was completed. Certain actions in the practices needed to take place (e.g. addressing queries following an audit), yet how they happened varied. Leadership for the work was key in this respect. In some cases, PNs assumed a central role, whilst others were only involved in a marginal way. Likewise, data show a tension in the importance placed on education for different healthcare staff, and whether it is multi- or single professional. It could be argued that it would be better to have a forum where discussion about managing HF took place between GPs and PNs, reflecting calls for attempts to change practice to adopt a multidisciplinary focus [12, 25]. However, as our data implied, PNs may be reticent to receive education with GPs because of their concerns about expressing uncertainty and knowledge gaps.

Overall, HFSNs had a high degree of clinical credibility and were an important part of GM-HFIT’s ‘facilitative unit’, alongside KTAs. Their specialist role enabled them to recommend changes in patient care based on guidelines; tensions due to professional boundaries did not appear to transpire or prevent GPs from accepting their recommendations. HFSNs served as a valuable interface between primary care and specialist services, and this appears to be an important role to develop in the future. HFSNs may initially lack contextual knowledge about how primary care operates and the heterogeneity of patients seen. Their improvement work at the interface of different sectors should therefore be supported by those with expertise in service delivery and knowledge mobilisation. Seconding non-specialist PNs is an alternative option, but they may be less knowledgeable when suggesting practice changes for a specific condition due to their lack of expert status. Further research is required to explore the importance of professional credibility in facilitation work within primary care.

### Strengths and weakness of the research

This study adds to the current literature by describing an intervention that had a strong evidence base with an explicit focus on context-tailored facilitation. It contributes to the broader knowledge mobilisation literature by demonstrating that facilitating change is shaped by a number of contextual tensions, which unfold at different levels. It also highlights the role of HFSNs as credible agents of improvement who can successfully bridge the gap between specialist and generalist care, when supported by knowledge mobilisation experts. Although the study was conducted in one region of England, its findings are likely to be theoretically generalisable to a wider range of clinical settings that deploy context-sensitive facilitation as a method of enhancing the uptake of research evidence.

Rigour was addressed by recruiting individuals representing all aspects of involvement in GM-HFIT, from across the three locations where this intervention was delivered. In addition, more than one person was involved in the analysis, which used an approach that allowed for consistency in how data were treated. Reflexive notes were made during data collection to document contextual information relating to where interviews took place and to record emerging ideas relating to the analysis.

We were reliant on individuals agreeing to talk to us. Variation in those interviewed was sought but it is possible people who held the programme in high regard took part. That said, we did receive comments about areas for improvement from participants. In this research we wanted to focus on the experiences of clinicians involved in the direct delivery of patient care. Future research might explore the views of administrative staff in GP surgeries as findings implied they could be important in maintaining an intervention such as GM-HFIT.

## Conclusion

GM-HFIT was developed to promote better management of patients with HF in primary care. Our data suggest that those attempting to facilitate change need to consider and understand context and associated motivations for involvement, which can be affected by micro and macro level priorities. Our study emphasises the need for a range of skills, possibly located within a team rather than an individual. The role of HFSNs, in particular, was identified as key in providing credibility when recommending changes to primary care staff. Although findings are based on a HF-targeted intervention, information about the manner in which context shaped facilitation and the tensions that emerged from differing internal and external influences probably extend to other attempts at implementing change within primary care.

## Abbreviations

HF: Heart failure
PN: Practice nurse
GP: General practitioner
HFSN: Heart failure specialist nurse
KTA: Knowledge transfer associate
QOF: Quality outcomes framework.
